# Cautious optimism: public voices on medical AI and sociotechnical harm

**DOI:** 10.3389/fdgth.2025.1625747

**Published:** 2025-09-23

**Authors:** Beverley A. Townsend, Victoria J. Hodge, Hannah Richardson, Radu Calinescu, T.T. Arvind

**Affiliations:** ^1^ York Law School, University of York, York, United Kingdom; ^2^ Department of Computer Science, University of York, York, United Kingdom; ^3^ Microsoft Research, Cambridge, United Kingdom

**Keywords:** public perspectives, medical devices regulation, AI-enabled medical devices, healthcare, medical AI, sociotechnical harm, socio-ethical and cultural requirement, prosocial values

## Abstract

**Background:**

Medical-purpose software and Artificial Intelligence (“AI”)-enabled technologies (“medical AI”) raise important social, ethical, cultural, and regulatory challenges. To elucidate these important challenges, we present the findings of a qualitative study undertaken to elicit public perspectives and expectations around medical AI adoption and related sociotechnical harm. Sociotechnical harm refers to any adverse implications including, but not limited to, physical, psychological, social, and cultural impacts experienced by a person or broader society as a result of medical AI adoption. The work is intended to guide effective policy interventions to address, prioritise, and mitigate such harm.

**Methods:**

Using a qualitative design approach, twenty interviews and/or long-form questionnaires were completed between September and November 2024 with UK participants to explore their perspectives, expectations, and concerns around medical AI adoption and related sociotechnical harm. An emphasis was placed on diversity and inclusion, with study participants drawn from racially, ethnically, and linguistically diverse groups and from self-identified minority groups. A thematic analysis of interview transcripts and questionnaire responses was conducted to identify general medical AI perception and sociotechnical harm.

**Results:**

Our findings demonstrate that while participants are cautiously optimistic about medical AI adoption, all participants expressed concern about matters related to sociotechnical harm. This included potential harm to human autonomy, alienation and a reduction in standards of care, the lack of value alignment and integration, epistemic injustice, bias and discrimination, and issues around access and equity, explainability and transparency, and data privacy and data-related harm. While responsibility was seen to be shared, participants located responsibility for addressing sociotechnical harm primarily with the regulatory authorities. An identified concern was risk of exclusion and inequitable access on account of practical barriers such as physical limitations, technical competency, language barriers, or financial constraints.

**Conclusion:**

We conclude that medical AI adoption can be better supported through identifying, prioritising, and addressing sociotechnical harm including the development of clear impact and mitigation practices, embedding pro-social values within the system, and through effective policy guidance intervention.

## 1 Introduction

Medical purpose software and Artificial Intelligence (“AI”)-enabled technologies (“medical AI”), with rapid expansions in the subfield of machine learning, show enormous promise to transform healthcare and the practice of medicine [1, 2]. Such technologies function to democratise expertise, automate drudgery, optimise resources, and push frontiers in healthcare [3]. While of far-reaching potential benefit, medical AI raises a set of important social, ethical, cultural, and regulatory challenges [4]. Although developed and deployed under varying global regulatory and governance frameworks, the regulatory approval of medical AI is typically framed in terms of patient safety risk, clinical performance, and device efficacy. Far less attention is directed towards the socio-ethical and cultural factors, and non-trivial sociotechnical harm and risk of harm, informing such approval and adoption [5].

Technology operates in ways that is influenced, at least in part, by the social and cultural context of its construction and is shaped by the values and beliefs of its creators and users [6]. As medical AI does not exist within a vacuum, but is embedded and integrated within a wider context of real-world societal and cultural practices, a commitment to responsible and ethical technological innovation calls for the consideration of the socio-ethical and cultural components of its adoption and the policies and ideologies that shape it [7, 8]. This is to say, responsible medical AI adoption should focus on clinical and technical safety and device efficacy, as well as novel forms of harm.

Our efforts here centre on “sociotechnical harm”, which considers both harm due to an AI system’s malfunction and internal failures that prevent it from operating as intended (i.e., technical harm), and harm resulting from the interaction between an AI system that operates as intended and the social and human contexts in which it is deployed [9]. As AI-enabled systems are composed of both technical and social properties, sociotechnical harm looks beyond the technical specifics or capabilities of an AI system to include social context, human interaction, ethics and cultural values, and systemic impacts. This includes an undertaking to identify, assess, and prevent injurious, adverse, or harmful consequences to those persons, groups, and communities for whom the technology is to serve. While a person’s relationship with the technology may drive its social value, harm extends beyond the person and their individual interests to broader collective harms to the group or wider society, such as instances, and the concomitant effect, of discrimination and bias on the community at large. Incorporating the perceptions, concerns, and opinions of diverse members of society - including minority and marginalised groups - enables AI stakeholders (such as AI designers, developers, and regulators) to understand and obtain an increased representation of culturally diverse and minority worldviews and the challenges they face in the deployment of AI. Rather than determining a priori what the normative concerns are, this work advances the perspectives and opinions of affected peoples and communities regarding normative issues and harm emerging vis-à-vis the implementation of medical AI situated within their real-world experience.

### 1.1 Sociotechnical harm

We describe “sociotechnical harm” with regard to medical AI broadly as any adverse, negative, or ill effect or impact experienced by a person or the broader society as a result of medical AI activity in the real world, lived experience of the person, member of the community, sub-group, or wider society [9]. This would include activities that cause iatrogenic harm and unintentional adverse outcomes (be they clinical, cultural, or social) resulting from medical interventions, applied for purposes of this paper, to instances of medical AI adoption. As such this refers to negative impacts (such as injury, loss, damage, and distress) and includes not only unfavourable health outcomes but all outcomes having an unfavourable physical, psychological, social, cultural, and ethical impact as a result of the AI-enabled medical adoption. The concern here is not only about how the technology fits within society and the environment, and its integration into clinical settings, but about its ill effect, directly or indirectly, on individual and societal interests, values, and well-being. For the most part, our focus is on harm that, unbounded by the technical system itself, emerges from the interrelationship between persons and the technology, and is, in nature, contextually dependent, relational, and socially embedded [10]. Although increasingly ubiquitous, sociotechnical harm often manifests in small and intangible forms that can be aggregated across applications and escalate into far greater and widespread harm [11]. Harm, in this context, spans domains and degrees of severity ranging from slight social, ethical, and cultural concern to more serious violations of human rights, freedoms, and civil liberties. The related term “risk of sociotechnical harm” refers to the probability of such harm occurring under defined circumstances. This work seeks to understand such harm as it relates to diverse peoples, escalates or diminishes, and evolves over time and throughout the entire medical AI lifecycle.

### 1.2 Research focus

Our analytical focus shifts from one that is top-down to empirical AI ethics that turns to the user group and research participants themselves to better understand what users of the technology consider to be desirable and undesirable practices (and of concern to them) at an individual, group, and societal level. Accordingly, the framing is as follows. First, we establish participants’ (be they users of the technology or potential patients) specific socio-ethical and cultural expectations and requirements with regard to medical AI development and adoption. Second, we consider sociotechnical harm, often overlooked by current AI knowledge, assurance, and regulatory processes, by establishing whether and how these technologies are perceived to be harm-causing to participants and their sub-groups and communities. Sociotechnical requirements and harm, although distinct are nevertheless interrelated. The exploration and integration of sociotechnical requirements at the medical AI design stage informs and seeks to prevent downstream sociotechnical harm. For instance, a robotic triage system that does not recognise that a user is hard of hearing, or has a speech impediment, or speaks with an accent might lead to sociotechnical harm. Although a growing corpus of literature has developed to classify harms across different technologies and applications [9], we looked to our research participants to provide localised and relevant anticipated harms as envisaged or drawn from their lived experience. Underpinning this is a growing vision to democratise technology by including wider public dialogue, engagement, and deliberation in technological innovation and regulation [12, 13]. Thus, in the face of sociotechnical harm, we engage with a diversity of stakeholders and community members who are epistemically better placed to hold knowledge and provide insight into the nature of the harm landscape [10]. Constructed through the lens of human-centric AI development, a broad set of concerns emerge from the use of medical AI in clinical contexts arising from the interrelationship between the human user and the technology itself.

Medical AI is governed by different types of existing regulation, such as medical device regulation, privacy laws, consumer rights laws, medical negligence laws, and health-related laws and policies with certain individual issues and concerns already well-documented. However, this study makes a contribution by capturing those types of harm that might fall out of scope of these instruments (avoiding system over-reliance, user alienation, or being treated without empathy or disbelieved, for example). Accordingly, it considers whether there is merit in augmenting the medical devices regulatory landscape to achieve increased identification and protection against a wider range of possible sociotechnical harm as indicated by public participants from minority or marginalised groups.

### 1.3 Public perspectives to inform policy development

This work is premised on the recognition that, however well-intentioned, AI-enabled technologies may lead to harm and that sociotechnical determinants of risk are crucial to safe medical AI adoption [14]. A UK government report demonstrated that the UK public has higher demands for AI governance in areas that are deemed higher risk or more complex, such as in healthcare.^1^ To that end, public perspectives involving persons’ and communities’ real-world lived experiences and the sociotechnical harms they identify can guide effective policy interventions, while prioritising the most pressing harms. These are harms that often run parallel to existing forms of social exclusion and marginalisation in society [15, 16]. Diverse and inclusive user-public input enables better integration and acceptance of medical AI in healthcare practice and serves to affirm the legitimacy of AI development in alignment with norms of responsible research practice [17]. Moreover, public distrust in technology is a significant barrier to adoption and is negatively associated with societal well-being [18, 19]. As foreground bearers of harm, a better health future involving medical AI is dependent, in large part, on clearly articulating and accounting for the perspectives of minority and marginalised persons and groups. Marginalisation is an active process that describes how, based on a person’s or group’s identity, they experience the world and how others perceive them in it [20]. Readily explored forms of marginalisation stem from racism, ageism, sexism, ableism, and classism [20]. Those experiencing marginalisation, often face structural forms of social exclusion and are disproportionately more at risk of sociotechnical harm from novel technologies [9].

### 1.4 Positioning

Previous studies have focused on AI in healthcare based on benefits, challenges, methodologies, and functionalities [21]. In addition, research has examined inclusivity [22]; iatrogenic disease [23] and algorithmic harm [11]. Further research has been conducted on social, legal, ethical, and cultural considerations and barriers to medical AI adoption [5, 19]; a review of the perceived threats posed by the usage of AI tools in healthcare on patients’ rights and safety [24]; in the domestication of AI tools in real-world care settings [25] and on perceptions and the perceived effects of AI in healthcare and health research [26–29]. Other studies have considered AI and social bias, such as ageism, sexism, and classism, and on AI and global justice and fairness, more generally [30–33].

This study underscores the importance of obtaining users’ perspectives in relation to technology adoption with a specific underlying emphasis on sociotechnical harm, impact, and user aspirations with regard to medical AI adoption. The research provides the opportunity for the collaboration between researchers and members of the public, allowing them to contribute to these rich and important conversations. Elicited through a process of participatory research, public perspectives provide a sound evidence base for responsible future innovation and can offer legitimate, thoughtful, and practical co-produced recommendations for implementation by policymakers [34, 35]. The realisation of this is dependent upon participant input to uncover, not only what the pressing sociotechnical harms and socio-ethical expectations and requirements are, but how this knowledge can be mobilised and applied by policymakers and translated into practical guidance. In a process of knowledge exchange, valuable learnings inform a body of justifiable evidence that can influence sociotechnical policy development. As attitudes to medical AI adoption evolve dynamically over time and across locations, it was important to capture as many perspectives as practicably feasible from diverse sources, as applied to diverse contexts. Although we refer to the term “public” in this paper, we acknowledge that no uniform public viewpoint or interest exists and that various groups, communities, and members of the public have, and will continue to have, different attitudes, beliefs, and concerns with regard to medical AI adoption.

## 2 Methods

**Figure 1:**
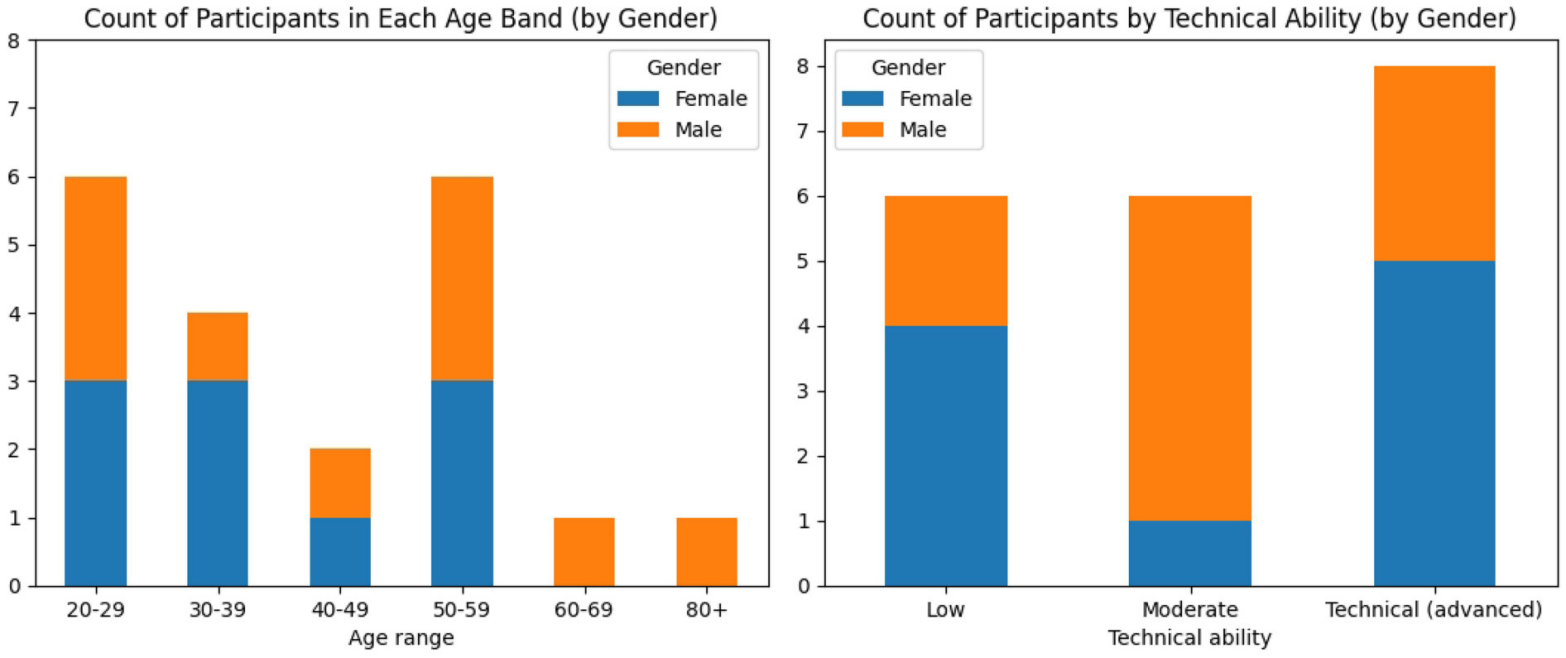
Charts showing the participants’ ages also broken down by gender (left) and participants’ technical abilities also broken down by gender (right).

Twenty participants (P1–P20) took part in the study of whom n=10 identified as female and n=10 identified as male. The age of the participants was quantised into 10-year age bands and ranged from 20–29 to 80+ years old as shown in Figure 1 with no correlation between age and gender.

Designing for inclusion ensures input from those persons and groups for whom the technology has the potential to inflict the most sociotechnical harm. The participants self-reported their level of technical ability with n=8 reporting advanced, n=6 moderate and n=6 reporting low technical ability as shown in Figure 1. There was no correlation between age band and reported ability and no correlation between gender and reported ability. In the support of diversity and inclusion, participants at high risk of inequality due to medical AI use were selected, that is, those that either reported protected characteristics in terms of the UK Equality Act of 2010 or had self-identified characteristics that although falling beyond the remit of the Act were nevertheless at risk for exclusion or inequality. 17 (85% of participants) self-identified as falling within at least one minority or marginalised group, including (n=10) females and (n=7) males. These included the non-exhaustive categories of (n=5) race, (n=8) gender, (n=1) sexual orientation, (n=1) age, (n=5) ethnicity, (n=3) religion, (n=7) disability/health condition, (n=4) education level, and (n=2) carer. Certain participants had unique requirements, displaying specific underrepresented criteria, such as monozygotic twins, neurodivergent, wheelchair user and persons living with colour vision deficiency (colour blindness).

Of the three participants who did not identify as marginalised, n=2 reported advanced technical ability and n=1 reported moderate ability, and all three identified as male.

Shifting the analysis to include marginalised, underrepresented, and minority groups allowed us to reflect a different set of important sociotechnical vulnerabilities and harms. We adapted the questions as appropriate for individual participants. Although a small sample will not be representative of a community or a region as a whole, we aimed to attain diversity in perspectives. Participants were purposely selected, that is, the sample was selected with the specific purpose or objective of maximising the range of views obtained.

### 2.1 Data collection

Using a qualitative design approach, semi-structured interviews and, at the participants request, questionnaires were completed by participants from September to December 2024 (see supplementary files). The questions were adapted to ensure appropriateness for the context and the participants, and anonymised questionnaires, notes, and interview-transcriptions were analysed. Participants were invited to voluntarily participate in the study and interviews were conducted in person or online and, if preferred, participants were invited to complete a questionnaire. Participants were provided with a privacy notice and an information sheet explaining the purpose, approach, and dissemination strategy of the research and a consent form was provided to participants for completion.

### 2.2 Research questions

Questions were used to initiate and guide the discussion and were centered primarily around socio-ethical and cultural expectations and sociotechnical harm with regard to medical AI adoption. An outline of the questions is provided in the supplementary material. The terms “sociotechnical concern” and “sociotechnical harm” were described to the participants and participants were encouraged to ask for clarification if terms and concepts used in the questionnaire and case study were unclear or unknown to them. Participants were encouraged to freely provide their perceptions, opinions and views related to the questions. Audio-recordings were transcribed verbatim and participant names were replaced with a number before the transcripts were analysised.

### 2.3 Data analysis

Data were analysed by BT and VH using a qualitative descriptive approach. The data analysis consisted of a process of data preparation, exploration, analysis, and interpretation following a Thematic Analysis Method [36]. From the contextually laden, subjective, and richly detailed data, themes and patterns were identified and arranged into core themes and emergent sub-themes. Coding and theme development progressed organically, in a manner that was exploratory and inherently subjective and involved reflexive researcher engagement [37]. Themes were conceptually developed at an early stage in the analytic process, drawn heavily from existing literature and theory. Characterising these themes reflect data related to a core, shared, meaning and capture the essence of recurrent meaning across the dataset. Themes, constructed from codes, were developed in the analysis so as to unify disparate data points [36]. This allowed us to remain close to the data and to the actual words used by the participants encouraging the description of participants’ ideas and views while inductively interpreting responses and grouping them into themes [38]. The data were collected, transcribed, organised, cleaned and coded by BT, VH, and HR. Review and coding of transcripts and questionnaires stopped when inductive thematic saturation was achieved and additional coding and thematic analysis would not result in any new codes or themes. The analysis of transcripts and questionnaire responses was undertaken once identifiers had been removed and de-identification manually assured.

The thematic analysis identified five key thematic categories:

• Theme 1: Sociotechnical concerns and harm types
• Theme 2: Trust and harm prioritisation
• Theme 3: Value identification and realisation
• Theme 4: Responsibility
• Theme 5: Regulatory oversight and policy intervention

### 2.4 Case study

Participants were encouraged to think of Medical AI applications and the impact and potential harm these applications might have on themselves and members of their groups. For purposes of illustration and positioned as a thought experiment, we described to the participants DAISY [39], a prototype “Diagnostic AI System for Robotic and Automated Triage and Assessment” of Emergency Department (ED) patients that we co-developed in a collaboration between the University of York, and York and Scarborough Hospitals NHS Foundation Trust in the UK.

Currently undergoing a feasibility clinical study at Scarborough Hospital, DAISY is a semi-autonomous, sociotechnical AI-supported system that interacts with consenting ambulatory ED patients by guiding them through a sophisticated triage process. This process involves the collection of four categories of medically relevant data for the ED patient being triaged: (i) demographic data—patient information such as sex and age, and their medical history (e.g., allergies, vaccinations and chronic conditions); (ii) anatomical data—specific parts of the body affected; (iii) subjective data—symptoms reported directly by the patient; and (iv) objective data—the patient’s vital signs (e.g., temperature and blood pressure). The first three categories of data are provided by the patient by means of a tablet computer, in response to prompts from DAISY, whereas the objective data are measured using a series of medical devices connected to the system.

Given these patient data, DAISY uses a suite of AI techniques to identify potential maladies, suggests further investigations (e.g., x-rays or blood tests) and patient referrals for assessment by specific hospital departments. Preliminary findings are then approved, amended, or rejected by the practitioner to facilitate the early stages of triage. DAISY links patient characteristics, demographics, and symptoms, viewed through the patients’ objective vital signs, to possible clinical states and to urgency and early treatment options. The benefit of the system is in the rapid categorisation of patients by severity, identification, and escalation of the critically unwell patients—and the generation of suggested investigation plans for subsequent approval by the practitioner. Practitioners can thereby streamline the early elements of the process to allow for additional treatment time and more effective resource management in critical cases. The study builds on previous work using DAISY as a case study undertaken to explore UK medical practitioners’ perspectives on medical AI adoption [19]. Further details about our ED patient triage system are available in the DAISY [39] clinical study summary.

## 3 Results

### 3.1 Cautious optimism

**Figure 2:**
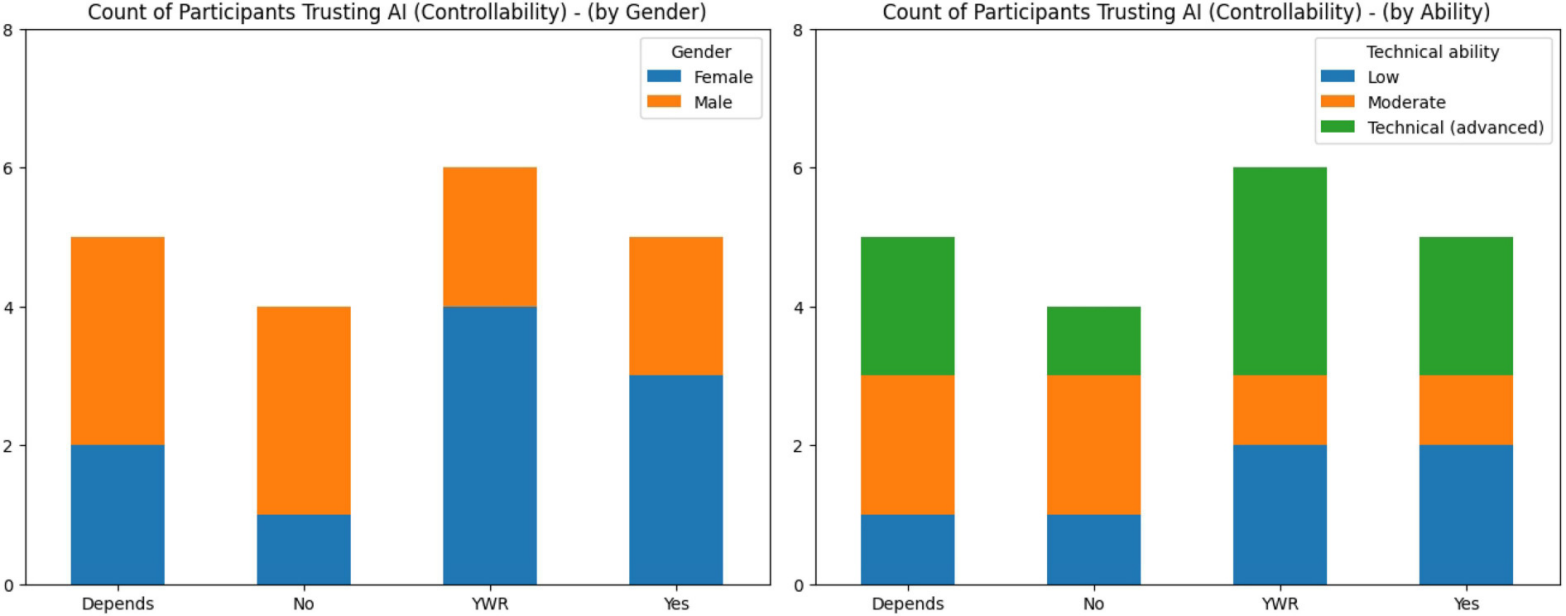
Charts showing the participants’ trust in medical AI and controllability broken down by gender (left) and technical abilities (right). YWR is “yes with reservations”.

While certain participants held strong views and distrust of AI, the dominant position was one of “cautious optimism”. Figure 2 shows the participants’ trust in medical AI with one chart broken down by gender and another by technical ability. Across genders and technical abilities, participants cautiously trust medical AI with only 20% (n=4) not trusting it while 55% (n=11) trust it with reservations or trust it sometimes but not other times. All participants (n=20) identified at least three potential sociotechnical harms with regard to AI adoption. This “cautious optimism” was described by P12 as follows: “*[Although] I am somewhat concerned about the use of medical AI; I believe that the benefits of these technologies can outweigh the potential risks*”. Overall participants believed that to harness the benefits of AI, adoption should be measured, pragmatic, and fair. While medical AI has the potential to increase accuracy, speed, efficiency in healthcare, it was believed by P14 that its increasing social impact required “*effective regulation in high-risk areas and fair public access to its benefits*”. Concern was expressed by P18 about the rapid adoption and transformation of medical AI with no clear understanding of how it will influence persons and their communities: “*…at a deeply human level, the technology risks further eroding our connection to our biological and emotional realities. Historically, technological progress has often outpaced our ability to anticipate or mitigate its unintended consequences*” with “*[t]echnologies that promise convenience often [coming] at a cost to our well-being*”. Optimism was tempered by the view that with far-reaching technological advancement comes the potential for adverse consequences and harm. P14 stated: “*The speed of development vs. effective regulation is a key concern …[together with] the potential consequences in terms of individual autonomy, social interactions and possible abuse by governments and the tech industry*”. While the majority of participants were optimistic about medical AI, many believed safeguards and assurances were needed to support its adoption with P4 stating: “*We need guarantees that no harm will come to us*”.

Broadly, the dominant position of cautious optimism aligns with the expectation that members of the public typically balance benefits in efficiency, speed, and accuracy of diagnosis with concerns around AI trust and safety. Themes of eroded privacy, compromised transparency, diminished equity, and impaired ethical alignment and safety correspond well to current AI ethics and governance literature [11, 40, 41]. However, what was less expected was the articulation of the subtle and more nuanced forms of social and cultural harm and various emerging themes. In particular the emphasis on epistemic injustice, the risk of alienation, the feeling of “otherness” and social exclusion, and challenges arising from emulated empathy and deception demonstrate unique risks to members of minority and marginalised groups in medical AI adoption. This clearly shows that harm is not perceived only as technical and clinical, requiring the necessary technical solutions, but also as social, relational, and culturally-positioned, requiring sociotechnical solutions. The following now sets out in more detail the nature of the perceived harms and various harm-types.

### 3.2 The nature of sociotechnical harm

**Figure 3:**
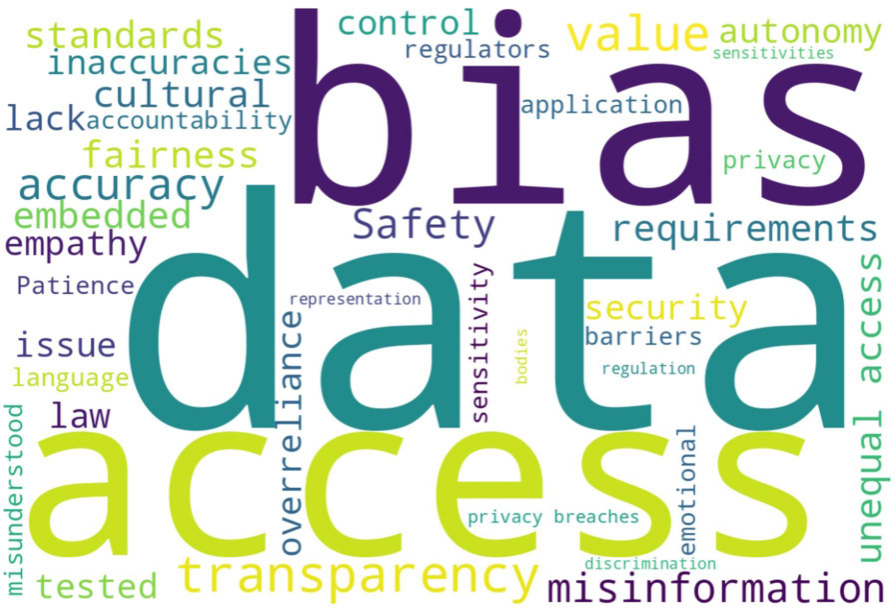
Word-cloud derived from the amalgamated participant response summaries (short-form free text) with stopwords and a small set of common words excluded. Common words excluded include: “may”, “might”, “will”, “use”, “system”, “systems”, “AI”, “medical”, “used”, “healthcare”.

Participants raised questions about how responsive the technology would be to their needs, and to those of their group and community, and whether the technology would cause harm. All participants were concerned about sociotechnical harm. Figure 3 shows a word cloud derived from the participants’ short-form responses when asked to list the potential sociotechnical harms of medical AI. Note: the responses did not include “trust” as the participants were asked specific questions regarding “trust” so it is not present in the word cloud.

The conceptualisation and severity of potential or hypothetical medical AI-related harm differed between participants. Ensuring safety was identified as significant with P17 stating that: “*Safety is the absolute priority in order to not harm patients with wrong outputs*”. Further statements were: “*[For me, i]mmediate harms are misuse and misinterpretation*” and “*medical AI carries greater risk of harm [as it] concerns the health, welfare, and treatment of potentially very vulnerable individuals*”. P6 emphasised that: “*It is important that AI considers my social and cultural requirements and my personal circumstances and experiences*”. Harm tended to focus on core harm-types, including: inaccuracy, safety and misdiagnosis, lack of human autonomy or oversight, being misunderstood or having limited ability to use the technology, data privacy and data compromises and breaches, issues around value and cultural alignment, access and equity, explainability and transparency, epistemic injustice, fairness, bias, and responsibility.

A priority was equitable access and treatment with P18 stating that: “*Equitable distribution of benefits is fundamental*”. Usability and interoperability were highlighted as of concern. P17 stated: “*It is important also to consider the usability making it as easy as possible for elderly people and [to] consider special adaptions in the design for people that need them*” and P16, “*I am concerned about interoperability and how these systems will fit within the entire healthcare supply chain*”. Significant harms were identified as equitable access, identified by 75% of participants (n=15); addressing bias and discrimination, identified by 55% of participants (n=11); and accuracy, identified by 55% of participants (n=11). Other harms included misinformation; data privacy and security; adaptability to social and cultural sensitivities and requirements; transparency; and safety.

### 3.3 Key issues for minority groups

Key concerns centered around furthering and deepening the “digital divide”, and about access and fairness. P20 cautioned that: “*I am worried about the technology divide leading to less access for marginalised sections of the community. For example, obtaining access to medical practitioners will be through digital apps which will require connectivity to access*”. Further key minority specific concerns were that: “*I think these systems can and should be made available to all*” and “*[I believe that] a fair and just AI system would feature no bias*”. Many participants believed that certain members of their communities would not have access to these technologies with P15 stating: “*Some members of my family and community will find this difficult and be excluded*”. The view was that equitable access to medical AI should “*enhance inclusivity, not exacerbate existing inequalities*” and should not according to P18 be “*limited by geography, economic status, or social position*”. Accessibility was a key concern across participant minority types: “*[These technologies] should be made accessible for all, not just the technologically literate or those of a certain age group*”.

Disconnect and alienation were identified as issues for minority and marginalised persons: “*I see alienation (and therefore missed opportunities for enhanced well-being) as a key risk for members of minority groups with protected characteristics (sex, race, religion, disability, neurodiversity) if the medical AI fails to provide assurance on essential matters—for example, confirmation that the AI has been trained and tested using diverse, non-discriminatory data or is otherwise adapted to accommodate the social or cultural norms of the group*”. On data as an aspect of harm: “*If minority groups are not properly considered in the data used for training and testing [the system], then the AI output clearly has the potential for harm and reduced efficacy for those under-represented groups*”.

### 3.4 Sociotechnical harm types

#### 3.4.1 Exclusion and access

Exclusion was a recurring concern and was identified as the most pressing harm. A key issue identified by the participants was whether medical AI would only be available to, and designed for, a specific profile of person. An overriding perspective was that exclusion would be inevitable and access unequal: “*Access will be unequal; many people will be excluded’,‘[p]eople will be excluded, it is inevitable*” and “*the majority of the people in my community will be excluded due to learning difficulties*”. While participants suggested that greater access to these technologies could help more persons to obtain healthcare, medical AI is seen to be elitist, available only to a select, privileged few. Inclusion criteria for accessibility were identified as the youth, those with technological prowess and capability, those comfortable using novel digital technologies, and affluence. With the evolutionary nature of medical AIs, participants excluded at the early stages of adoption feared a widening gap or inability to “*catch up*” over time. This also speaks to the idea that the digital divide might be exacerbated by further marginalising existing groups, or by the creation of new groups based on the artificial creation of different and novel exclusion criteria and identities.

The failure to take into account personal circumstances was seen as an exclusionary factor. Exclusion for reasons of technical literacy, system complexity, language barriers, financial constraints, apprehension (fear), and age were well documented in the study. On digital literacy: “*[t]hese systems will] exclude those who are not technically competent. If the system is simplified, I would be better able to use it*”. P4 indicated that they would like to see language, accent, and skin colour addressed. On reasons for exclusion, P11, P13, and P15 contributed that “*the way we do things in our culture will make using these systems difficult for me*”, that “*[i]t is important that [the system] is able to recognise my language, the way I speak, my accent, and that I am dyslexic*”, and “*[s]ometimes I cannot understand [human] doctors and nurses when they are talking to me, let alone them understanding me. So, I do not see why it would be any different for a robotic system*”.

Cost was seen as a constraint, potentially precluding access to those vulnerable and most in need of access to healthcare in certain communities. Regarding financial constraints, the belief was that for many participants access to medical AI would be unaffordable, costs would be high, or require expensive equipment or software, such as access to smartphones, with P11 stating: “*…the majority of the people in my community will be excluded because we cannot afford [to use or purchase the technology]*”. On the lack of education and apprehension informing exclusion: “*[Exclusion] can occur as …the system seems difficult to use or could be intimidating*” and: “*it might exclude people who are fearful of technology*”. Participants were concerned that older persons and those with special requirements might avoid use: “*I fear that older generations who are seriously less tech savvy would struggle to read the screens given the font size and/or use the touchscreen as this is new to them*”. Again, on technical literacy, P5 stated: “*Technical literacy is a problem. Those who are disabled such as the visually impaired (as no braille version exists) will be excluded without support*”.

Exclusion and a reluctance to use the system may result from specific requirements not being met. Specific requirements reported by P3, P9, P13 and P17 including amongst others that: “*I have performance anxiety when things are unfamiliar to me, so I will experience discomfort*”; “*I have sensory deficiencies—how will the system manage this?*”; “*Expression in English is often difficult for me. Will it understand me, and will I understand it?*”; and “*[t]he system may mishear (or misinterpret) something I say*”. Not accounting for personal features or circumstances was seen to be problematic, P1 stated: “*Facial recognition systems erroneously allow me to open my identical twin’s mobile phone and have access to his banking app, imagine this in a healthcare context*” and P4: “*as I am a new mum I am likely to have different and unique requirements at the moment*”.

Related to exclusion were the themes of access and equity. Participants were concerned about fair distribution and access to medical AI often reported as a pressing concern: “*If the system is good and works well then everyone should have the ability to use it, otherwise it becomes divisional and a wealth lottery. The idea behind the NHS is that healthcare should be fairly distributed—so should medical AI (again, if it is effective and trustworthy in what it does). This could even fill the gaps and help to make [healthcare] more fair and equal*” and, pointedly, “*[a]ccess concerns are significant. Inequities in access could deepen existing disparities, leaving marginalised communities further behind*”. Participants also expressed views on prioritising access and directing access to those most in need: “*I think it is important to prioritise access to those most in need, therefore I would not be concerned about not having access as I likely would not need to be prioritised*”.

#### 3.4.2 Bias

Not only can AI systems produce prejudicial outcomes, but they stand to replicate and amplify patterns of general systemic and structural negative or unwanted bias within society, such as racial-, age-, and gender-related bias and discrimination [15, 16, 42, 43]. Negative bias in the model may contribute to worse health outcomes for certain population groups and is understood to be a significant contributor to health inequality, exacerbating and entrenching health disparities [44]. For purposes of this study, we refer to “bias”^2^ as the difference in performance or treatment between persons or subgroups compared to others for a predictive task [45]. Participants used the term “bias” largely pejoratively. “Discrimination”^3^ is referred to as either direct, that is, the treatment of a person less favourably than another because of a particular protected characteristic or attribute (such as, age, gender assignment, sex, sexual orientation, disability, marital status and so on), or indirect, that is, when a condition, practice, or policy that applies to everyone disadvantages persons who share a protected characteristic. These protected characteristics closely align to the study criteria used for inclusive and diverse participant selection. P18 stated the following: “*[A]s a gay immigrant, I can be treated in discriminatory ways, whether overtly or subversively*”. The risk of such unwanted bias and discrimination transferring to the system was not lost on the participant. P2 stated: “*My biggest concern is that these systems are biased and discriminate against certain groups, potentially caused by a lack of interdisciplinary design teams with a range of expertise and (life/domain) experiences*”. The implications of bias were highlighted further by P2: “*[I am concerned about] bias going unnoticed until it causes significant enough issues and then [that] the resolution may be to “fix” that specific symptom rather than the root cause of the problem*” and, importantly, by P9 who stated: “*I am worried that algorithms can widen the gap and further polarise society*”.

Participants commented on fairness and data bias in the context of potential sociotechnical harm: “*[I believe] a fair and just AI system would feature no bias*”, “*[I am worried] that systems built on biased data may reinforce societal inequalities, affecting employment, healthcare, and access to services*” and “*[d]ata bias for gender is a concern as predominantly male data is collected and not much data relates to women and pregnant women in particular. I think [women and pregnant women] are not represented, or are under-represented, in the [training] data*”. The concern is that: “*[Models], trained on data, will reflect that data and if not representative, this can lead to bias and [the model] not performing well [or not at all] for certain populations*”. P20 commented: “*[An issue for me] is bias in data leading to worse outcomes for individuals with unusual conditions or a complex set of co-morbidities. Difficult and unusual cases will, by definition, be less prevalent in the data used to train systems*”. Lastly, on transparency around the identification of bias, P19 stated: “*I would absolutely like to understand the sources of bias in the system or application’s design*”.

#### 3.4.3 Data privacy and data-related harm

Health-related data often constitutes sensitive, personal information about a person and the use, processing, and sharing of data was discussed. Infringements on data protection rights and data-related harms, such as data breaches and data security, were highlighted as of pressing concern. The following was cited by P12, P9, P16, and P2: “*Medical AI carries greater risk of harm due to dealing with sensitive personal information*”; “*I would like to know what personal (and other) data is used and for what purpose [and] who has access to it and how long it will be kept for. Especially in medical contexts as the data may be very sensitive, embarrassing, or dangerous*”; “*I am concerned about the security and confidentiality of my data*”; and “*[m]y concern is that if a system and data security is not prioritised, the system can be abused, misused, or hacked [and my concern is that of] lack of control over data and privacy*”. On a specific issue about data quality and integrity, P1, an identical twin, remarked: “*in my experience with facial recognition it picks up both of us…I would like to be sure that [the system and the data used are] able to tell the difference…[What if] it brings up the wrong person’s records. They could give me the wrong medication or wrong diagnosis*”.

P2 and P10 commented on the importance of data security and on sharing data: “*Avoiding unauthorised access to the information is important [to me]*” and “*if it helps to improve the system then I would share my data but I should be made aware of this and give consent*”. Concerning training data and the composition and provenance of datasets, P4 stated: “*That is the biggest risk of all. I am very worried about the misuse of data and the [accuracy] of training datasets*”. Further comments were: “*[I am] concerned about the quality and representativeness of the data used to train the algorithm, or generative AI, [and where it] comes from sources which are not the most trustworthy or reliable (or are simply outdated)*”; “*I don’t mind my data being used to train medical AI and for research, but not for profit or commercial purposes*”, “*I am concerned that AI could be used for harm and to get my data*”. In addition, a related data comments by P18 was: “*I would not be happy if my data was scraped from the internet without my knowledge*” and P3 was concerned that control of data and the algorithm might be outside of their local community or country.

#### 3.4.4 Iatrogenic harm and safety issues

Harm resulting from inaccuracy, misinformation, and the lack of safety of the medical AI was identified as significant. Iatrogenic harm refers to any unfavourable response or harm experienced by a patient resulting from medical treatment [23, 46]. As the risk of such harm exists in traditional medical practice, P6 suggested that it will likely transfer to medical AI scenarios: “*[medical AI] will still make mistakes, but then again, there’s always human error as well, so you cannot always account for everything*”. The vast majority of participants were concerned about unintentional or consequential harm resulting from medical AI treatment. Misdiagnosis, inaccuracies in treatment and data, under-performance or unreliability of the system, and safety were seen to be the source of potential harm: “*I am concerned about misinformation and data inaccuracies*”, “*harm to me means being wrongly diagnosed and misunderstood*”, and “*I am very worried about reliability, accuracy, and safety*”. Without safety assurances and testing, P9 believed medical AI implementation is premature and worrying stating: “*I am concerned—I do not believe that the quality of AI is particularly sufficient or reliable (especially not in the medical sphere!)*”. Concern extended to physical harm, with P11 stating: “*I am worried that a [robotic] system may act violently [towards me] and cause me physical harm and I will not know how to stop it*”.

#### 3.4.5 Human autonomy and human oversight

Participants were concerned that human control and human autonomy would be eroded and that they might give up control to misaligned AIs. Encapsulating these sentiments were opinions such as: “*It is important for me to have some level of control or autonomy over the functioning of the [medical AI, that is,] to be able to opt in or opt out at any time*”. While human oversight was seen to be important, relinquishing control was, to some extent, seen to be an inevitability. P6 pragmatically stated: “*It would worry me if the system made decisions on its own without a human to oversee it, but that is the point of having the system, not so?*”. The level of oversight should, however, depend on the nature of the application, the context, and degree of risk involved. P18 suggested that: “*In critical domains like healthcare, [I believe] a human should always be in the loop to ensure accountability and contextual understanding. For lower-stakes applications, automated systems might operate independently, but there should still be clear mechanisms for human oversight and intervention if errors occur*”. P4 was of the view that: “*I think some single decisions can be made without human oversight*”. More specifically, concerning autonomy and consent, P7 stated: “*If as a carer I am acting on behalf of another, I would expect the system to have the necessary checks in place to ensure I can act for someone else, like I would have to in a situation involving human doctors*”. Certain healthcare functions, such as lower risk administrative tasks like scheduling appointments, for instance, lend themselves more easily to AI involvement, however, “*actual engagement with the patient requires more oversight and human involvement.*” The opinion was expressed that limitations should be placed on capabilities with P6 “*not wanting AI to do everything*”. Perspectives were divided over independent AI decision-making. While certain participants would trust the system to make decisions, others believed a human health professional should review output: “*I think that any recommendations made by the medical AI should be reviewed by a healthcare professional before a decision is made regarding the patient*”. One clear suggestion was that users be told when AI is involved in clinical decision-making: “*I should always be informed when AI is involved in decision-making processes*”.

#### 3.4.6 Alienation and standards of care

Diverse populations with different ways of acting, relating, and narrating, often in vulnerable states, have varying social and cultural requirements and expectations for medical AI [19]. The social consequences of medical AI were highlighted including that its adoption may diminish the healthcare experience, change the relationship between doctor and patient, or profoundly influence how we relate to medical practitioners, each other, and ourselves. The rapid advancement of AI is seen as a challenge “*at a deeply human level, where the technology risks further eroding our connection to our biological and emotional realities*”. Questions were posed about how medical AI mediates relationships with human medical practitioners and human carers, and what it means to be human and be treated as a human, with interconnection, loneliness, and vulnerability as central issues. The lack of human connection and a lowering of the qualitative experience of healthcare, and a “*human touch*”, were seen as ways to increase alienation and to diminish the quality and standard of care. P13 stated: “*talking to artificial intelligence gives [me] a sense of isolation*” with P18 stating: “*AI amplifies disconnection*”. The same participant expressed concern that “*by prioritising efficiency and virtual interaction, we risk losing touch with fundamental aspects of our humanity: our physicality, our need for face-to-face connection, and the slower, grounding rhythms of nature*”. P18 went further: “*This disconnection is not just an abstract concern — it could have far-reaching consequences, for example, the more we detach from our biological reality, the more our capacity for empathy, emotional regulation, and meaningful relationships may erode*”. The suggestion was that: “*Without deliberate safeguards and a focus on aligning AI development with human needs — such as fostering connection, preserving neurobiological balance, and supporting emotional well-being — we risk creating a future that is not only unsustainable but profoundly inhuman*”. The under-explored relationship between the use of non-human, artificial technologies and their effect on unwell and vulnerable human patients was repeatedly emphasised. In this regard, P9 stated: “*the most vulnerable members of our society (like old people) must be protected*”. That these technologies will change the very fabric of society and interact with us at our most vulnerable moments was seen to be deeply troubling to certain participants. P16 posited: “*I feel medical technologies, generally, are very invasive and intrusive*” and “*as recent diagnostic procedures made me feel intruded upon, I can only imagine how intrusive these systems will be*”.

#### 3.4.7 Emulated empathy

Emulating empathetic care, the role of emotional engagement, the inability to relate to human frailties, or to relate with sensitivity, dignity, and compassion to users, as well as the risk of deceptive practice were identified as of concern. The requirement to protect the vulnerable was repeatedly emphasised, P14 stated: “*[We must] protect and not mislead vulnerable patients regarding trained AI bots simulating empathy vs. the practical therapeutic benefits of AI bots for certain patients when human resources and family are unavailable*” with P13, on the value of empathetic care, stating: “*[I believe] we should try to incorporate empathy in some way [and] to try to not increase patient stress levels. At the same time it is important to inject values which ensure [that] different cultures, beliefs and orientations [are represented]*”. An expressed perspective was that certain experiences may be difficult to relay to a non-human system, as P9 stated: “*Because of past trauma, it is hard for me to speak about certain topics*”. However, P2 and P5 saw this as a potential benefit: “*There may be less …judgement compared to speaking with a human*” and “*I do not want to disclose certain things to a human doctor for risk of being judged*”. Further, ongoing exploration is required into the conceptual distinction between what it means for systems to be empathetic (i.e., be sympathetic to the feelings of users), and empathic (i.e., able to read feelings accurately). Most participants were doubtful that the former is, or will ever be, possible with P9 stating: “*I would find it hard to believe that [the system] can provide empathetic care*”. The participant continued: “*Certain things [the robot] will never understand—sad and critical situations, tragic situations, and abuse, for instance*”. Whereas persons may engage with medical AI well understanding its limitations, they nevertheless minimally expect to be treated with certain prosocial values in mind, that is, gently, humanely, and with dignity.

#### 3.4.8 Over-reliance and dependency

Over-reliance and dependency on medical AI outputs were seen to be of concern. A participant expressed the opinion that: “*[Overreliance] may be an issue where people become less thorough and meticulous with their work if assisted by AI*”. A further view was be that: “*Over-reliance on medical AI might diminish the notion of individual responsibility for personal care, and responsibility for caring for others in your community*”. P14 stated: “*A key concern …is uninformed over-reliance by the public*”. “*Understanding a system’s limitations*” and “*managing expectations*” were offered as solutions.

#### 3.4.9 Epistemic injustice

Epistemic injustice emerged from the data as a key sociotechnical harm. Epistemic injustice refers to forms of unfair treatment that specifically relate to issues of knowledge, understanding, and participation in communicative practices [47]. It is the act of being unfairly treated in your capacity to know something by means of, inter alia, exclusion and silencing; systematic distortion or misrepresentation of your meanings or contributions; and undervaluing your status or standing in communicative practices. Epistemic injustice presents when a person is not believed, and their credibility is unfairly reduced or dismissed, based on the way they speak or present their views often because they are of a particular ethnicity, gender, age, or socioeconomic class. As incidences of epistemic injustice are often heightened in minority and marginalised groups, the risk that epistemic injustice may be carried through to, or perpetuated by, the AI technology was raised: “*I feel that I have not been believed because I am a young person. I would be worried if the system does this, and I expect the robotic system to be better in those situations*”, “*I am very worried that the system will find my stories less credible*”, and “*[human] doctors have previously disregarded my statements where I signaled that I was stressed or anxious, will this happen with AI-enabled technologies?*”. Epistemic injustice may be introduced where the system does not have the capacity to “receive” a person’s testimonies and therefore ignores, overlooks, or dismisses them [48]. To this end, participants expressed concern that the system might dismiss their views, opinions, and testimonies: “*I am concerned that the system may not have the capacity to understand my stories*”, “*I feel like the system will dismiss my voice or opinion because of the way I speak, or the way I am, [or] because I speak with an accent and my English is not good*”, and “*[my view is] that often women are seen as less credible and one would hope this will not be translated over to a medical AI system*”. Similarly, a concern around understanding was that: “*The system might take everything [I say] literally*”. Importantly, a related issue was that epistemic injustice might result if persons are afforded no or limited opportunity to describe or comfortably express themselves: “*[I believe a] drawback is that it is more difficult [for me] to assert [my] case with a robot than with a human*”. A further concern was that medical AI may perpetuate epistemic injustice learned from training data: “*If the AI system is learning from historical data, it might introduce this [type of] injustice*”. P4 advanced the opinion that if mitigation measures were introduced, epistemic injustice could be reduced: “*[I am] sometimes not taken seriously due to my gender, particularly in medical contexts, for instance, when listing certain symptoms to GPs. I think that AI systems could help to correct this, but only if human bias is not programmed into the system through training data, for instance, [or by being] instructed to dismiss certain comments, or to make assumptions based on gender*”.

#### 3.4.10 Deception and transparency

Transparency was seen as presenting an additional layer of complexity for medical AI adoption. For all participants knowing that they were engaging with an AI system—or being the product of algorithmic decision-making - was very important. Many participants believed they had a right to know or to be told, specifically in cases of doubt or uncertainty. Overwhelmingly, the expectation was that: “*Patients [users] should always be told when AI is used*” and “*I would want to know if AI was involved and whether I was dealing with a robotic system*”, “*I would want to know that I am talking to a machine*”, “*I would want to get feedback and to be able to dig a bit deeper into whether in fact the system understands me*” and “*[t]ransparency is the most pressing issue in my opinion and the government is responsible to regulate this*”. Awareness is believed to be important where medical AI relates and interacts with users and invites user reactions: “*I would like to see full disclosure. If I am a patient and I am engaging with some sort of AI, I need to be made aware of the fact that I’m not engaging with a human, and that I’m engaging with a machine. And I think it’s just fair for all parties to be on the same departure page. In terms of who’s delivering care? Who’s making the decisions? And what human involvement oversight there is vs. the control and autonomy you give the AI system*” and “*[I would like to be] made aware of the risks of utilising AI—bias, incorrect information etc—[so that I can] decide for myself instead of having this choice taken from me which could be considered paternalistic. If a user is not told this [it] could be a type of deception as they could believe they are speaking to a person when they are not*”. P1 expressed the view that: “*[Without transparency, these technologies] might be weaponised or politicised without our knowledge*”.

Participants indicated that they would want explanations communicated to them: “*I would like to know how [the system] came to the decision it made, on what basis the decision was made, and what information it used*” and “*[i]t is important to me that the system is able to explain why something took place the way that it did*”. However, P5 believed that not all information should be disclosed and that information should be reasonable and appropriate: “*It is important that the information provided is appropriate. Some information should not be provided, and certain information like sensitive information must be filtered through a human. Not unfettered transparency, I do not want to hear serious diagnoses like cancer from an [AI-generated] report, this is likely to cause me more distress and to be damaging to my mental health*”. P5 continued: “*[t]here is harm in disclosing prematurely or inappropriately, especially without human, empathetic support*”. Expectations about explainability and transparency were: “*[I would like an explanation] if answers, recommendations or decisions are given or made, then how and why these came about. [Also] what information is being used and how this is stored [and] reassurance that data is being protected and securely stored (and potentially deleted after a certain amount of time or when no longer required by the institution who collected/stored/used the data) and not sold on*”, “*[i]t is important [for me] to know the background and where the technology comes from*” and “*I would like to know of any political or economic incentives or other factors driving its development and adoption*”. One participant went further: “*Transparency is critical. I would like full disclosure of the data sources used to train the AI…and any significant influences—whether political, economic, or cultural—behind the technology’s design and implementation should be openly shared*”.

### 3.5 Value identification and operationalisation

Value alignment and the possible integration of prosocial values within the system were canvassed. 90% of participants (n=18) cared about value and cultural alignment, with certain participants questioning how this might be achieved in practice. Two participants were unsure of what value integration might mean practically and how it could be reasonably implemented. P9, in favour of prosocial value integration, stated: “*As a minimum, these technologies should have appropriate values embedded within them*” and that this should be a “*top priority*”. P4 indicated that *Rules and Values should be embedded’*. P2 stated that “*I care about safety, fairness, the protection of my private data, [that] AI systems should not physically or mentally harm me or cause significant and unnecessary stress, confusion, or concern*”. P13 commented that values were context dependent, questioning whether there was one sense of right and wrong and: “*what values would be put in?*”. More pragmatically, and with a view to aid this integration, P5 suggested that: “*Practical things can help like being invited to a summary page or confirmation page, for example, to make me feel I have a say and am empowered to check my responses and confirm any input*” or “*[r]eassuring me that my input is confidential and that my data is lawfully processed and will not be shared with others*”. P10 suggested that inclusive measures such as *’simple and user friendly interfaces’* might assist. Such practical operational measures to introduce and embed value at the design stage of the development process may alleviate certain aspects of sociotechnical harm. Prosocial values identified as important were: “*respect, equality, dignity, diversity, patience, gratitude, thoughtfulness, unbiased opinions, gentleness, empathy, personability, and humour*”. It was suggested by P20 that “*[f]oundational values should be applicable across the domain and application types, [but] getting everyone to buy into them may be more difficult*”. However, P10 and P14 questioned whether introducing value into the system could easily be achieved: “*How do we standardise values when we live in a pluralistic culture?*”, with P10 opining: “*I am absolutely certain that values, laws, and principles should be practically and operationally, incorporated and implemented within the system and that this should be a top priority of those developing these systems. However, I can see opposition to this. I believe there are strong arguments over ‘what values?’ and what laws or principles should be incorporated. Regardless, I believe that there must be some form of values and laws implemented that can capture a general sense of societal value*”.

### 3.6 Responsibility

Although participants differed in opinion about who should be held to account, one view was that: “*For me responsibility means the manufacturers, designers, developers, regulators—a number of parties, it’s a shared responsibility*”. Certain participants suggested that “*responsibility comes from everyone*”, with P11 adamant that end-users should “*never be held accountable*”. On regulatory responsibility, one view was that: “*I would feel better if the system was endorsed by a particular certified body, like the medical regulators*”, however P10 expressed the view that they would not necessarily trust a system deemed “trustworthy” or “certified” by a regulator as it would “*depend on the regulating body*”. A lack of clarity and recourse to hold those responsible and to challenge decision making was identified as problematic. P9, P13, P16, P17, P19 and P20 believed that governance should be achieved by “*[a] combination of industry self-regulation, strict laws and compliance, and embedded values in the technology*” and “*international standards*”. P14 expressed the view that: “*…tech companies who design, develop, train and test the AI systems, their authorised representatives and funders, the entities which deploy [bring] AI systems to the market, governments, and the regulators who fail to maintain standards*” should share responsibility. Attributing responsibility was seen as important “*for keeping people safe [and for] preventing future harm*”. P20 offered a possible solution: “*We live in a diverse society where the views of individuals are not homogenous. As such, deriving a single pathway for care, whilst efficient, may well favour sections of society inappropriately. Having a set of pathways which are appropriately devised to cater for these cultural needs will ensure great acceptance across cultural divides*”.

### 3.7 Regulatory oversight and policy mitigation

The role of regulation, regulatory oversight, and the public’s participation in informing regulation were explored. 85% (n=17) believed regulation of medical AI systems is necessary and 90% (n=18) believed that sociotechnical harm should be addressed and prevented by regulatory policy. Inasmuch as regulation and oversight were considered to be critical to medical AI adoption, questions of how this ought to be done, and avenues of recourse to identify and address harm, were frequently posed. As medical AI grows in adoption and complexity, and with the novel issues emerging from, for example, the advancement and adoption of generative AI, regulatory oversight was identified as of particular significance. P13 believed that oversight and a clear understanding of the risks and impact of medical AI are needed with: “*Countries adding regulations to existing standards*”. P20 opined that harm is inevitable in healthcare but “*acceptable bounds*” on harms should be made explicit and procedures defined to monitor these harms with “*appropriate reporting strategies*” as part of the regulatory approval. An opinion was that regulatory approaches should be sensitive to risk and application type, and to the context within which the medical AI was to be deployed with P2 stating: “*Not all systems carry the same risk of harm, and some [applications] are far more risky than others and should be regulated differently*”. Lack of testing and oversight were raised as issues, in the words of P8: “*these things need to be thoroughly tested*” and of P2 and P18: “*Regulation [must be] stricter with medical technology than with other applications*”, with clear methods for assigning responsibility “*should things go wrong*” and for “*holding those accountable*”.

#### 3.7.1 Trust and role of regulatory authorities

Most participants trusted regulatory authorities to “*do the right thing*”, although trust was often conditional, for example, P2 indicated: “*[I trust the regulators, but] this requires constant review of existing systems and of the way in which the regulators oversee and assess the technology*”. Whereas certain participants stated they would trust UK regulatory authorities and organisations such as the NHS, others were far more cautious. P18 stated: “*Regulators often struggle to keep pace with the rapid advancements in AI technology, and their assessments may overlook biases, contextual gaps, or longer-term implications. This erodes my confidence in their ability to fully assess these systems’ ethical and practical impacts*” and “*[c]ertification from a trusted regulator would provide reassurance, but I’d still approach it critically*”. Insufficient understanding of adverse long-term societal impacts post-deployment and the possibility of downstream implications were described. P6 questioned whether there may be any “*aftereffects*” or “*adverse effects*” and worried about things going unnoticed or things that may not be anticipated. Opinions about who should be authorised to regulate medical AI were also articulated. P20 suggested: “*[Medical AI should be regulated by] an independent central agency. This might well be governmental but should absolutely be independent of the technology providers*” and P18 on what should be examined: “*I would like regulators to examine bias and fairness (and that AI systems don’t disproportionately harm or exclude specific groups); that I am offered clear reasoning behind decisions; that my personal data is kept private and secure; that outcomes are validated in diverse, real-life scenarios; and that long-term implications are monitored as [these technologies] evolve and have societal effects*”. P5 stated: “*We need to understand how to detect in which instances a device will not work at all, or in a limited way, for an individual or a group*”.

#### 3.7.2 Public participation in policy determination

If failure to address user requirements, expectations, and aspirations can lead directly or indirectly to sociotechnical harm, addressing this harm can, in turn, transform medical AI into AI for good. One way of supporting this is through regulatory intervention. Perspectives were divided on whether members of the public should inform or influence regulatory policy. P13 said: “*I do not think I, or a random member of the public, have enough understanding to influence the development of AI in the medical field directly*”. Participants believed that participatory engagement in the regulatory and design process should be supported and promoted: “*There should be active engagement with users in order to understand their life experiences, requirements and expectations*” with the expectation that “*[r]egulators need to be far more inclusive and participatory with regard to what the public wants and their expectations*”. P1, P3, P4, P5, P9, P10, and P19 felt that being “*heard*” and “*having a collective voice*” were important: “*I would like a voice in what is important, but it is a broad community collective response, not necessarily an individual one*” and on informing the regulatory process: “*I would like to have a voice [to determine] what is important*”. P16 went further stating: “*[A]s a taxpayer and citizen [in the UK] I would like to have a say, or at least be informed. Participation and inclusion is important and should be increased*”. P2 was of the view that: “*Regulators should include assessments of user engagement and feedback in regular audits to ensure this is taking place*”. Diversity and inclusion were emphasised with the requirement to involve wider communities and real-world, lived, perspectives: “*Regulatory approval should account for diverse socio-cultural contexts to ensure the AI serves all communities equitably. Neglecting these considerations risks creating tools that exclude or harm specific groups*” with P18 adding that: “*[r]egulation should be inclusive, involving diverse stakeholders, including patients, marginalised communities, and experts from various disciplines. This ensures that the regulation addresses real-world needs and reflects societal values, rather than being driven solely by corporate or institutional priorities*”.

## 4 Discussion

### 4.1 Empirical ethics and value realisation and alignment

This study forms part of a growing call to ensure that medical AI adoption is safe and effective, and foregrounds public good. There is recent awareness of the value and contribution of research involving public participants in advancing safe and responsible AI use and policy development, and as a means to uncover conceptual and practical ways of identifying and managing harm [8]. Whereas clinical safety and efficacy are fundamental to safe and responsible medical AI adoption, we show how social, ethical, and cultural normative values can emerge from interactive processes involving participant users. We use empirical data as descriptive evidence to account for these important emergent normative claims and, more practically, in the prevention and mitigation of sociotechnical harm. An example of this is the emerging harm about epistemic injustice and the provision by participants of possible solutions to overcome some of these difficulties. These findings support and reinforce the importance of inclusive, participatory voices in traditional AI policy interventions.

Integrating empirical accounts with the practical operationalisation of prosocial values is one way of making these systems more supportive of sociotechnical goals and of reframing and addressing harm as a way to derive good. Yet, the alignment of medical AI systems with embedded norms, prosocial values, and societal interests is largely under-explored. Understanding the context-dependencies into which the medical AI is to be deployed speaks to the idea of human-centric AI design as a key framing which positions human values, thoughts, and experiences central to the discussion. It grounds the technology with individuals and human aspects, allowing us to establish, first and foremost, what works well for humans and society [49]. Accordingly, our efforts interrogate human values and are oriented towards human outputs that are socially and culturally located in an attempt to understand what users value and to support them in the realisation of this value [50]. The results of this study can inform value-based guidance and critical future policy development and allow us to think more seriously about medical AI design that can operationalise values and address and mitigate harm.

### 4.2 Regulatory reform and development

Medical AI present unique sociotechnical challenges. The study shows that 100% of the study cohort were concerned about sociotechnical drivers of harms in medical AI adoption, with 85% looking to the UK regulators to address these concerns. We need, therefore, to understand the existing regulatory position and to consider paths to sociotechnical reform. In the UK, medical AI is regulated by the Medicines and Healthcare products Regulatory Agency (“MHRA”). For medical devices to be brought to market they must meet the requirements of the UK Medical Devices Regulations 2002 (as amended).^4^ The MHRA also provides guidance and policy for developers to follow, including guidance on the regulatory approval for testing and evaluating the safety and effectiveness of medical AI. In line with the UK’s proposed flexible and pro-innovation approach to AI adoption, the MHRA is embarking on a pragmatic programme of regulatory reform for medical devices^5^ through high-level, general requirements. This sectoral approach advocates for an incremental, light touch approach to regulation [51]. Given the number of issues at hand, and aligned with the already published work of the International Medical Device Regulators Forum (IMDRF),^6^ the World Health Organization (WHO),^7^ and the U.S Food and Drug Administration (FDA),^8^ the MHRA has set out early guidance on medical AI,^9^ including setting out how the five principles contained in the UK AI White Paper^10^ can be applied to medical AI. In addition, the British Medical Association recently set out principles for AI and its application in healthcare.^11^

Medical devices and medical AI are governed by, amongst others, existing medical device regulation, privacy laws, intellectual property laws, consumer rights laws, medical negligence laws, and health-related laws and policies including a number of existing standards and guidelines for the development of medical devices. In the United Kingdom, this will also involve undertakings and related roles to assess and prevent sociotechnical harms shared between various regulatory agencies, offices, and services—including, the Medicines and Healthcare products Regulatory Agency (MHRA),^12^ the Information Commissioners Office (ICO),^13^ the UK Office for AI, the National Institute of Health and Care Excellence (NICE),^14^ the Health Research Authority (HRA),^15^ the General Medical Council (GMC),^16^ and the National Health Service (NHS).^17^ However, there is no single, structured medical AI regulatory awareness framework that explicitly identifies and assesses sociotechnical risk of harm across medical AI design and development processes. Moreover, a fragmented regulatory landscape with insufficient redress mechanisms for medical AI harm might mean that sociotechnical harm and risk of harm proceeds unchecked. In addition, highly consequential longer-term, future social and cultural impacts have significant yet largely underexplored implications.

The development of medical AI technologies and the practical adoption of standards and guidelines requires integration into existing design processes and regulatory frameworks. While worldwide standards address certain aspects of sociotechnical harm include, for example, ISO Standards on Fairness (ISO12791 and ISO5339); on Transparency (IS5339 and ISO38507); on Accountability (ISO38507 and ISO37301); on Risk Mitigation (ISO37301 and ISO12791) and on Societal Impact and Ethical and Societal Concerns (ISO38507, ISO5339, and ISO24368), we should consider more carefully how these fit within existing risk management frameworks, how gaps identified in the findings can be addressed and aligned, and their wider regulatory implications. In the case of evaluating sociotechnical harms, one option for developers would be to integrate into their existing risk and quality management practices for both medical devices and AI (such as ISO 14971, ISO 13485, ISO 42001). Here the possible sociotechnical harms may serve as additional prompts alongside established characteristics affecting safety.

### 4.3 Future directions: sociotechnical harm awareness and mitigation

When we look at the study data, sociotechnical harm takes various forms, levels of severity and risk, and impacts both individual persons and the wider community and society. Harm can be immediate or longer-term, and can be confined to particular individuals or have large-scale, downstream implications affecting society more generally, such as the erosion of trust or continued systemic exclusion from use. Moreover, reducing the risk of harm in certain areas might deepen harm in others by shifting harm elsewhere; and seemingly innocuous and intangible harm can be aggregated to cause far greater and differently-presented harm [11]. Accordingly, addressing sociotechnical harm requires a two-fold approach. In the first instance, it requires a more complete and integrated articulation of harm involving definitional clarity across the AI space. Levels of harm range from perceived individual to community and systemic risk of potential harm. Second, it requires improved harm detection, evaluation, correction, and value operationalisation measures. A requirement for both pre- and post-deployment evaluation can seek to investigate specific cases of sociotechnical harm and their impacts, including data privacy-related harms, iatrogenic harm, issues around bias and discrimination, increased alienation and exclusion, and epistemic injustice. This also involves, in combination, the articulation of commonly shared regulatory guidelines incorporating specific AI principles to address specific sociotechnical harm, tools and methods for mitigation and evaluation, the establishment of best practices and a set of disclosure obligations, the introduction of harm-centric impact assessments, mandatory audits, benchmarking procedures, and criteria for the selection of testing and training data, ethics review, and commitments to increased stakeholder engagement. As sociotechnical harm presents differently across settings and domains, with differing degrees of severity, an extended approach is required. Certain standardised but targeted interventions can help to identify, prioritise, and address, and importantly, minimise harm. Measures to combat sociotechnical harm might be applied throughout the stages of the medical AI lifecycle, be they at the design, data collection and management, model building and development, verification and validation, or monitoring and real-world performance evaluation stages. Doing so would necessitate a more granular understanding of the different types of sociotechnical harms with a view to argument-based assurance, remedy, and mitigation. It also offers measures that align with the degree of complexity, risk, and level of autonomy impacted, and are guided by ideas of proportionality and appropriateness.

To date, practical recommendations for the introduction of sociotechnical harm mitigation and reduction measures in medical AI development and adoption remain at an early stage of policy development [40]. The contribution of this study is to provide candid accounts of subtle and more obvious perceived harms in medical AI adoption for minority groups. This is fundamentally exploratory in nature offering rich and diverse qualitative insights. Future work should involve mapping and translating findings - which remain at a high level of abstraction - into actionable policy pathways and measures that apply to specific regulatory instruments. These can, in turn, lead to, and augment, specific new UK and EU AI regulations and medical AI policy initiatives.

### 4.4 Limitations

While the research cohort was drawn from the general population, with diversity and inclusion prioritised, its composition is biased toward those willing to participate in the study, given the time commitment and effort involved. The sample size of 20, although small, was diverse and carefully selected, providing comprehensive and nuanced insights. A diverse sample, albeit a small one, with sufficiently detailed data accuracy and richness can add significant perspectives to policy-making processes of engagement and can provide richly textured, experientially-valuable information [52].

The case study, positioned as a thought experiment, required certain participants with self-identified low levels of technical capability and with limited epistemological foundation to envisage the implications of a prototype AI-enabled medical system. This meant that perspectives may have been ill-formed or poorly articulated. We overcame this challenge, in part, by describing all technological terminology and processes in language that was appropriate and easily understood. In instances of uncertainty, participants were encouraged to seek clarification and further information. To ensure participants remained focused only on socio-ethical and cultural issues of adoption, discussions were guided around a set of pre-established core issues from which further emerging issues were generated. While discussions of risk of sociotechnical harm were centered on participants themselves, participants were also encouraged to think about the implications of medical AI and possible ensuring harm on family members and members of their communities and groups.

## 5 Conclusions

This study shows that the UK public is cautiously optimistic about medical AI adoption and concerned about sociotechnical harms associated with emerging and future medical AI technologies. Based on a qualitative design approach with 20 members of the UK public, drawn from racially, ethnically, and linguistically diverse groups and from self-identified minority groups, a range of concerns were observed. These included, amongst others, privacy, the lack of human autonomy, the role of emulated empathy and epistemic injustice, and deception and transparency, as well as the need to maintain safety and effectiveness of the system. Key concerns were risk of exclusion, inequitable access, bias, and data-related harms. Further to this, the UK public expected regulatory authorities to play a role in addressing sociotechnical harm. This study makes an important contribution by centering minority voices in debates around medial AI adoption in support of the anticipation of harm. As there is limited data on the sociotechnical considerations and perceived harms of AI adoption in medical contexts today, this creates an opportunity to build evidence-led policy around how to plan for, and mitigate, such risks of harm. Harm awareness and mitigation frameworks can help to ensure that medical AI is better supported through identifying, prioritising, and addressing sociotechnical harm throughout the entire AI development lifecycle. Through the development of clear impact and mitigation practices, it is also possible to embed pro-social values within AI technologies, and to provide added policy support and guidance to AI designers and developers.

## Data Availability

The raw data supporting the conclusions of this article will be made available by the authors, without undue reservation.

## Footnotes

^1^https://www.gov.uk/government/publications/public-attitudes-to-data-and-ai-tracker-survey-wave-2/public-attitudes-to-data-and-ai-tracker-survey-wave-2 (accessed 04 February 2025).

^2^https://www.iso.org/standard/84110.html.

^3^https://www.legislation.gov.uk/ukpga/2010/15/section/13.

^4^https://www.legislation.gov.uk/uksi/2002/618/contents.

^5^https://assets.publishing.service.gov.uk/media/64a7d22d7a4c230013bba33c/Medical_device_stand-alone_software_including_apps__including_IVDMDs_.pdf (accessed 04 February 2025).

^6^https://www.imdrf.org/docs/imdrf/final/technical/imdrf-tech-131209-samd-key-definitions-140901.pdf (accessed 10 December 2024).

^7^https://www.who.int/publications/i/item/9789240078871 (accessed 19 February 2025).

^8^https://www.fda.gov/medical-devices/software-medical-device-samd/artificial-intelligence-and-machine-learning-aiml-enabled-medical-devices (accessed 05 February 2025).

^9^https://assets.publishing.service.gov.uk/media/662fce1e9e82181baa98a988/MHRA_Impact-of-AI-on-the-regulation-of-medical-products.pdf (accessed 05 February 2025).

^10^https://www.gov.uk/government/news/uk-signals-step-change-for-regulators-to-strengthen-ai-leadership (accessed 04 February 2025).

^11^https://www.bma.org.uk/advice-and-support/nhs-delivery-and-workforce/technology/principles-for-artificial-intelligence-ai-and-its-application-in-healthcare (accessed 17 February 2025).

^12^https://www.gov.uk/government/organisations/medicines-and-healthcare-products-regulatory-agency (accessed 25 August 2025).

^13^https://ico.org.uk (accessed 25 August 2025).

^14^https://www.nice.org.uk (accessed 25 August 2025).

^15^https://www.hra.nhs.uk (accessed 25 August 2025).

^16^https://www.gmc-uk.org (accessed 25 August 2025).

^17^https://www.nhs.uk (accessed 25 August 2025).
